# Translation of the Satter’s Division of Responsibility in Feeding Questionnaire into Brazilian Portuguese: A Cross-Sectional Study

**DOI:** 10.3390/nu15112575

**Published:** 2023-05-31

**Authors:** Rafaella Dusi, Raquel Braz Assunção Botelho, Eduardo Yoshio Nakano, Fabiana Lopes Nalon de Queiroz, Renata Puppin Zandonadi

**Affiliations:** 1 University of Brasília, Faculty of Health Sciences, Department of Nutrition, Campus Universitario Darcy Ribeiro, Brasilia 70910-900, Brazil; rafaella.souza@aluno.unb.br (R.D.); raquelbotelho@unb.br (R.B.A.B.); fabinalon@hotmail.com (F.L.N.d.Q.); 2 University of Brasilia, Department of Statistics, Campus Universitario Darcy Ribeiro, Brasilia 70910-900, Brazil; nakano@unb.br

**Keywords:** division of responsibility in feeding, Brazil, children, instrument, psychometrics

## Abstract

This cross-sectional study aimed to translate and perform a psychometric analysis (evaluation of reproducibility and internal consistency) of the sDOR.2-6y™ into Brazilian Portuguese. The translation and back-translation followed the protocol required by the NEEDs Center, and the approved version was called “sDOR.2-6y™—Português-Brasil”. The approved version was submitted to a test–retest round to verify its reproducibility through the Intraclass Correlation Coefficient (ICC). A pilot study was performed to assess the internal consistency of the instrument. The reproducibility analysis (*n* = 23) showed a total ICC of 0.945. With the data from the pilot study (*n* = 384), the internal consistency evaluation was analyzed through Cronbach’s alpha coefficient, and the instrument obtained an overall Cronbach’s alpha of 0.301. The translation of the sDOR.2-6y™ into Brazilian Portuguese is the first and only tool available for the Brazilian population to exclusively assess the division of responsibility in feeding, which is essential to the academic community, health professionals, and research on child feeding. Therefore, this instrument in Brazilian Portuguese will allow future research on the division of responsibility in feeding among those responsible for children in Brazil.

## 1. Introduction

Healthy eating is crucial to children’s growth and development in their early years [1]. Nutrition is directly related to a child’s health status. It affects biological, psychological, and social aspects, protection against diseases, and even childhood morbidity and mortality [2,3]. The relationship between children and food in the early years directly affects eating habits. Healthy eating children are more likely to become healthy adults with awareness and autonomy to make better food choices [3]. In this sense, a healthy child’s eating is related to parental feeding practices that favor the development of autonomy in the child’s feeding. Children are allowed and encouraged to express their preferences and to make choices among healthy foods available and pre-selected by their caregivers. It also encompasses stimulating the child to perceive and attend to their hunger and satiety signals to guide the amount of food they consume. Healthy eating by children includes the collective aspect of eating, and a safe and pleasant environment. Family meal consumption also impacts children’s nutrition, and exposing them to safe foods with positive examples has important impacts on healthy food consumption [4].

Recent changes in dietary practices have resulted in a visible increase in rates of excess weight in children, which affects the evolution of children’s health. There is a decreased consumption of healthy foods and an increased consumption of industrialized foods rich in sugars, fats, and chemical additives. In addition, due to the fast pace at which heads of households work, family members do not eat meals together as often as before and do not prepare home-cooked meals based on fresh food as often [3]. It is essential for children’s health to respect signs of hunger and satiety. Furthermore, it is crucial to observe the child’s rhythm of eating and to offer healthy meals patiently and in a healthy environment that promotes pleasurable eating. It is also important to establish limits for negotiations about food while still allowing the child to make choices and to restore the habit of eating as a family [3,4]. Some instruments have assessed parental practices or infant eating behaviors, such as the Comprehensive Feeding Practices Questionnaire [5], the Child Feeding Questionnaire [6], the Child Eating Behaviour Questionnaire [7], and the Feeding Practices and Structure Questionnaire-28, which assess parental feeding practices and mealtime data [8], but none of these instruments evaluate the division of responsibility in child feeding.

Child feeding depends on the skills of the caregivers and the child. It requires caregiver confidence in the signals provided by the child, thus dividing the responsibility for feeding [9,10]. The division of responsibility in feeding (sDOR) is an authoritative model proposed by Satter, and it is based on trust. It describes the role of the caregiver and the child in the feeding process and, based on these two central dimensions, details the functions of each one. In the sDOR model, parents are responsible for deciding what (what types of food will be provided), when, and where food will be offered. Children are responsible for deciding what (the child chooses from the pre-selected foods that parents have made available), how much, and if they will eat the food offered to them [9,11,12].

In Brazil, the Ministry of Health recommends actions that are consistent with sDOR, such as suggestions that caregivers choose healthy foods offered to children while also allowing them to choose from among the offered options; that the signs of hunger and satiety shown by the child should be respected; and that a pleasant environment favors the child’s agency at family meals [3]. The importance of following these principles is confirmed by studies that show that when children receive adequate support for the development of autonomy in feeding, it results in more varied choices, balanced caloric intake, and, consequently, expected growth and development [13]. This reinforces the importance of adequately assessing data on the Brazilian population’s confidence in the division of responsibility in feeding.

Due to the need to measure adherence to the sDOR principles and the inexistence of an instrument that exclusively measures this, the sDOR.2-6y™ was developed in English to be applied to the US population. Because it is a recent study, the sDOR.2-6y™ has only been evaluated and validated for the US population, despite having approved translations in Arabic and Danish [13]. It is a questionnaire with 12 items divided into five domains (1—mealtime structure; 2—what is available to the child; 3—how food is available to the child; 4—respect for child autonomy in eating; 5—who controls what, when, and how much is eaten) [13]. The total score ranges from 0 to 36; the higher the score, the stronger the parents’ adherence to sDOR. Scores above 24 represent good adherence to sDOR [13,14,15]. This instrument has been proven to predict nutritional risk in children. It can indicate caregivers who adopt behaviors compatible with responsive feeding practices, without using pressure to make their children eat, for example [13].

Despite the importance of the division of responsibility in feeding and the variety of other good instruments available, there is no instrument in Brazilian Portuguese capable of measuring all aspects of sDOR. Therefore, this study’s primary objective is to translate and perform a psychometric analysis of the sDOR.2-6y™ into Brazilian Portuguese. Translating and applying an instrument validated into other languages is interesting because the greater the use of the same instrument in different populations, the greater the possibility of making comparisons among studies and of gathering concrete results through research, in addition to saving time and financial resources in research [16,17,18].

Using an instrument in a country with a different language from that initially designated for it requires translation and cultural adaptation processes to make the instrument’s language culturally compatible with the target population without changing its concepts or content, thereby maintaining the validity and reliability of the original instrument [18]. Even in instruments that are already well consolidated, the importance of performing translation and cultural adaptation is explained by the fact that there are significant cultural differences between countries [19]. Therefore, this instrument in Brazilian Portuguese will allow future research on the division of responsibility in feeding among those responsible for children in Brazil.

## 2. Materials and Methods

**Figure 1:**
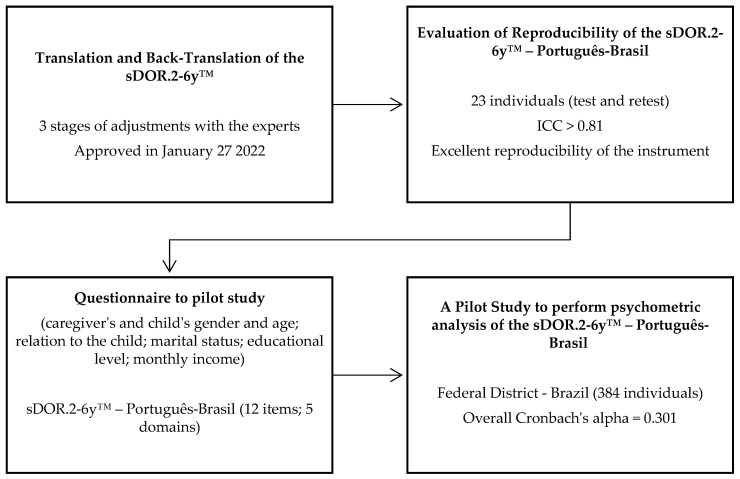
Flowchart of the steps to translate and perform the psychometric analysis of the sDOR.2-6y™ into Brazilian Portuguese (sDOR.2-6y™—Português-Brasil).

This cross-sectional study, approved by the Research Ethics Committee of the Faculty of Health Sciences of the University of Brasília, Brazil (CAAE 56301222.1.0000.0030), was divided into three steps: (i) Translation and back-translation of the original sDOR.2-6y™ to Brazilian Portuguese; (ii) Evaluation of reproducibility; (iii) A pilot study to perform a psychometric analysis of the sDOR.2-6y™ for the Brazilian population (evaluation of internal consistency). Figure 1 shows a flowchart of the steps to translate and perform the psychometric analysis of the sDOR.2-6y™ into Brazilian Portuguese.

### 2.1. Translation and Back-Translation of the sDOR.2-6y™

This step followed the NEEDs Center’s protocol [20], which is the instrument’s copyright holder. Other studies [21,22] have also performed the same translation process, which followed the NEEDs Center guidelines [20,21,22,23]. Two translators performed the first step of translating the instrument from English into Brazilian Portuguese with native Brazilian Portuguese language and without prior knowledge of the sDOR.2-6y. The translators were informed about the importance of the translation and of focusing on the concept, e.g., on the translation of the meanings of the items (not on the literal translation of the words); the need for the translation to use understandable language for people with a basic educational level; and the need to adopt gender-neutral language (use of neuter pronouns) to avoid confusion during application. The original instrument comprises twelve items: 1. My family has meals at about the same times every day; 2. I let my child eat whenever she/he feels like eating; 3. If I think my child hasn’t had enough, I try to get him or her to eat a few more bites; 4. When I am home at mealtimes, I sit down and eat with my child; 5. I struggle to get my child to eat; 6. I decide what foods to buy based on what my child eats; 7. I let my child feed him/herself; 8. I let my child eat until s/he stops eating and doesn’t want more; 9. I am comfortable with providing meals for my family; 10. I make something special for my child when s/he won’t eat; 11. I let my child have drinks (other than water) whenever s/he wants them; 12. We have food left over after meals [13,20]. The items were scored according to the NEEDs Center recommendations. Each item receives a particular score for each possible answer. The score table is released by the NEEDs Center to researchers after granting authorization to use the instrument. Therefore, the information can be obtained by contacting the NEEDs Center [20].

Each of the translators performed an independent translation, and a researcher familiar with the instrument analyzed these two translations. Through consensus, the researcher and translators arrived at a single version of the first translation step. The translated version was applied in a pre-test stage to a convenience sample of Brazilians (*n* = 5) with no knowledge of the subject of the sDOR.2-6y™. These participants were asked to explain what they understood about the meaning of the instrument’s items. Based on their comments, the translators and the researcher made adjustments and reached the final version of the instrument for Brazilian Portuguese (*translateBR)*. The *translateBR* was back-translated into English by a third translator, who did not participate in any previous stages and had no knowledge of the sDOR.2-6y™. The *translateBR* and its back-translation were sent to the NEEDs Center for approval. After minor adjustments, the approved version of the sDOR.2-6y™—Português-Brasil was authorized for application in Brazil [20,21].

### 2.2. Evaluation of Reproducibility of the sDOR.2-6y™—Português-Brasil

The test–retest of the sDOR.2-6y™—Português-Brasil was performed using a convenience sample to verify the instrument’s reproducibility through the Intraclass Correlation Coefficient. The inclusion criteria were being a Brazilian mother, father, or caregiver of children aged 24 and 72 months living in the Federal District/Brazil. Because of the need for two responses from the same person in two different moments, a convenience sample composed of the researchers’ contacts that met the inclusion criteria was necessary to control access to the same person to answer the instrument for a second time. The recruited participants did not know about the need to answer twice and were informed not to spread the instrument at this stage.

The instrument was entered into Google Forms© along with the informed consent form and questions about the participants’ and children’s age and gender, initial letters of the respondents’ first and last name, and the degree of relationship with the child. Considering that not all invited individuals would answer the instrument, a convenience sample of 53 individuals who met the inclusion criteria received on 21 June 2022 an invitation to answer the instrument (by the researchers’ personal communication through instant messaging or social networking applications). The aim was to reach a minimum number of 20 test–retest participants [24].

The agreement of responses in the test–retest evaluation was verified using the Intraclass Correlation Coefficient (ICC). Data were interpreted considering values of ICC below 0.40 as “poor”, between 0.40 and 0.59 as “fair”, between 0.60 and 0.74 as “good”, and between 0.75 and 1.00 as “excellent” [25]. The ICC was calculated using the two-way mixed effects model [26], assessing absolute agreement and considering the mean of the observations. Of the 53 individuals initially invited, 23 agreed to participate in both stages. To evaluate reproducibility (test–retest reliability), the instrument was resent to the same individuals. Participants were asked to answer it again, with a minimum difference in response time of 48 h and a maximum of 15 days.

### 2.3. A Pilot Study to Perform a Psychometric Analysis of the sDOR.2-6y™—Português-Brasil

After confirming the instrument’s reproducibility, we performed a pilot study to assess its internal consistency. This stage occurred between 6 July and 5 August 2022. It drew on a convenience sample of Brazilians who were fathers, mothers, or caregivers of children aged 24 and 72 months living in the Federal District/Brazil (inclusion criteria). Dietitians or students in nutrition courses were excluded in this study, as proposed by the original study [13].

The survey used the Google Forms© tool. It contained the informed consent form, the title of the study and its objectives, and information about the possibility of the subject refusing to participate and the confidentiality of the collected data. After that, the individuals who accepted to participate were asked about being nutrition students or nutritionists. If they answered affirmatively, they were automatically guided to the end. Those who went on to the following stages of the survey were directed to answer sociodemographic questions, questions about any specific children’s health diagnoses (such as food allergies, eating disorders, or autism spectrum disorders), and the sDOR.2-6y™—Português-Brasil items.

Participant recruitment was conducted using a snowball method via social media because this has been proven to be an effective and efficient way to recruit participants for research, allowing for a larger sample size, a shorter completion time, and reduced application cost [27,28]. The recruitment of subjects was active, with outreach on social media and through personal contacts of the researchers. People who received the link to the research were encouraged to spread it to their acquaintances who fit the target audience. At this stage, a minimum of 20 respondents were expected [29] for each instrument item in the internal validation process. Therefore, the minimum sample size was 240 participants, considering the 12 items of the sDOR.2-6y™—Português-Brasil [29].

### 2.4. Statistical Analysis

The obtained data were extracted from Google Forms© into a Microsoft Excel© spreadsheet, and the software IBM SPSS© (Statistical Product and Service Solutions), version 22, was used to analyze the data. The Cronbach’s alpha coefficient was calculated to analyze the instrument’s internal consistency, which is widely used in cross-sectional studies, such as the one proposed. It evaluates the degree to which the instrument items correlate with each other [30]. Descriptive data of the sDOR.2-6y™ scores were presented in terms of mean and standard deviation (SD), and the characteristics of the sample subjects were categorized and presented by frequency and percentages. The scores of the sDOR.2-6y™–Português-Brasil were compared with sociodemographic characteristics by independent Student’s *t*-test or one-way analysis of variance (ANOVA) with Tukey’s post hoc tests. The normality of the observations was verified through a Kolmogorov–Smirnov (with Lilliefors correction) test. A 5% significance level was set (*p* < 0.05) in all tests.

## 3. Results

### 3.1. Translation and Back-Translation of the sDOR.2-6y™

The final translation of the 12-item sDOR.2-6y™—Português-Brasil was approved by the NEEDs Center on 27 January 2022, after three stages of adjustments with the experts. The final approved version of the translation (sDOR.2-6y™—Português-Brasil) can be found on the NEEDs Center website [20], which authorizes its use only upon project submission to apply the instrument.

### 3.2. Evaluation of Reproducibility of the sDOR.2-6y™—Português-Brasil

Through the Intraclass Correlation Coefficient (ICC), the instrument’s reproducibility was evaluated in its five domains and in total. Table 1 shows the analysis results of the 23 respondents (mean age of 37 ± 6 y/o; 78.2% mothers) with a total Intraclass Correlation Coefficient of 0.945 for the sDOR.2-6y™—Português-Brasil. Observing the results by domains, the Intraclass Correlation Coefficient for all domains was greater than 0.81, except for the domain “Respect for child autonomy in eating,” which was 0.70.

### 3.3. A Pilot Study to Perform a Psychometric Analysis of the sDOR.2-6y™—Português-Brasil

In the pilot study, there were 384 valid responses. Table 2 details the participants’ characteristics. The caregivers participating in this study were mostly women (84.6%) and children’s mothers (82.6%). The average age of the studied population was 38 years (SD 5.36) (71.6% of the population was aged between 31 and 40 years old). They were mostly married (88.8%), had a high level of education (75.8% were graduates), and had a high family income (68.2% received more than BRL 10,000). The children about whom the caregivers answered the questions were mostly girls (52.1%), up to 5 years old (72.2%), and without any specific health diagnosis (79.6%). The internal consistency evaluation data were analyzed through the instrument’s Cronbach’s alpha coefficient. The sDOR.2-6y™—Português-Brasil obtained an overall Cronbach’s alpha of 0.301.

Table 3 shows the pilot study participants’ sociodemographic characteristics by the sDOR.2-6y^TM^—Português-Brasil domains. Except for the children’s gender in domain 5, in which male children presented higher scores than females, all the other results did not show a significant difference.

## 4. Discussion

This study carried out the translation, back-translation, and cultural adaptation of the sDOR.2-6y™ into Brazilian Portuguese. In addition, the internal consistency and reproducibility of the instrument were evaluated, and a pilot study was conducted to perform a psychometric analysis of the translated sDOR.2-6y™ with a Brazilian population. The process was strictly conducted following the instructions by the NEEDs Center [20], the holder of the instrument’s copyright, and is in line with the process described in other studies [16,18,19,21,31]. It is worth noting that this study is the first to perform a psychometric analysis of an instrument to measure sDOR using the Brazilian Portuguese language.

As described in the Guidelines for the Process of Cross-Cultural Adaptation of Self-Report Measures [18], using an instrument in a new country and in a language different from the one in which it was originally designed requires a translation process and cultural adaptation, as performed in the present study. The importance of this process is based on the need to make the language culturally compatible with the target population while maintaining the content and concepts presented in the original instrument [18]. This is because there are significant cultural differences between countries [19]. This process seeks to obtain equivalence between the newly translated instrument and the original, and to keep the validity and reliability of the original instrument. However, it is worth noting that this is not always the result obtained by translation and cultural adaptation, and this can result in changes in the psychometric or statistical properties of the instrument [18].

The advantage of translating an instrument that has already been validated in other languages and populations is the possibility of comparing data with other studies, optimizing research time, and saving research resources [16,17,18]. Another study points out that translation allows the usability of an instrument built in another language, reinforcing that it is a less expensive process than building a new instrument from scratch [32]. The cultural differences between nations, as well as the period in which the instrument is applied, also reinforce the importance of performing cross-cultural translation and adaptation even in already well-established instruments [19]. Using a validated sDOR instrument in different countries allows comparisons among different populations, helping health professionals and governments trace strategies, action plans, and public policies on child feeding specific to each country/culture.

The test–retest was performed to analyze the reproducibility of the instrument. The first response to the retest was received two days after the first test was sent, and the last response to the retest was received six days after the first test was sent. The retest was stopped when the number of subjects exceeded the recommended number of 21 subjects. The interval between responses is consistent with what is suggested in the literature, which states that the interval between responses can vary from hours to years [33,34] but indicates an interval between forty-eight hours and two weeks [33]. In the test–retest stage, a similar study that translated an instrument into Brazilian Portuguese showed time intervals between responses compatible with our study [21]. It is also worth noting that the literature states that the longer the interval between responses, the more the test–retest reliability will decline, because a significant time interval provides more chances for changes in the behavior of the assessed subjects [33,34].

The total Intraclass Correlation Coefficient of 0.945 for the sDOR.2-6y™—Português-Brasil indicates excellent [25] reproducibility of the instrument. Because this is a recent instrument [13], no studies have evaluated the reproducibility of other translations of the sDOR.2-6y™ in another language. All instrument domains obtained an ICC between 0.75 and 1.00, indicating excellent reproducibility [25], which means reliable measurement will be consistent in its values even if multiple measurements are taken [29]. The exception is the domain “Respect for child autonomy in eating”, which obtained an ICC of 0.7, showing good reproducibility [25]. The literature states that results closer to 1 show higher reliability of the instrument [35,36].

The fact that most of the participants in the pilot study were women can be seen as a gender bias, but this is consistent with other studies, such as a similar one conducted in the same region (which obtained about 56% of responses from women) [21]. Another translation and validation study conducted in a different region in Brazil obtained 75% female participants [37]. A more recent study conducted in Brazil with parents or guardians of children that assessed food neophobia also showed a population that mainly comprised women, about 86% [38]. The original validation study of the instrument also obtained significant female participation, where 94% of the respondents were women [13]. Because women are more concerned with health and children’s health issues, the populations in such surveys are expected to more likely be female [38,39].

Because this is an online participant recruitment survey, the population profile was expected. More developed areas tend to have better communication structures with internet access, and internet users tend to have higher levels of education and income [40,41].

Internal consistency analysis revealed relatively low Cronbach’s alpha values, but this is consistent with the original paper validation of the sDOR.2-6y, which found a Cronbach’s alpha of 0.32 [13].

The number of items [42,43,44] and dimensionality [43] affect the alpha value, and this must be considered when interpreting the values [42]. A low alpha value can occur due to a low number of questions, which may lead to underestimating reliability [43,44]. Therefore, due to the nature of the sDOR.2-6y™ (12 items divided into five domains), a low Cronbach’s alpha value was expected, as its value is directly affected by an instrument’s number of items and domains [42,43,44,45]. Some studies suggest no hard limit for acceptable or unacceptable alpha values, and even instruments with low alpha values can still be useful [46,47]. Because no other instrument exclusively measures sDOR [13], the importance of using the sDOR.2-6y™, even with low Cronbach’s alpha values, is evident.

Male children presented higher scores than females in the sDOR.2-6y™—Portuguese-Brazil domain 5, which deals with “Who controls what, when, and how much is eaten”. It is known that there is a difference in parenting styles between boys and girls. Girls generally receive more positive parenting, both from their father and mother. Therefore, boys are encouraged from a young age to be more independent than girls [48]. At mealtimes, boys are more fussy [49], and caregivers are more likely not to endorse emotional eating in boys [50]. Regarding feeding control, the impact on girls and boys is different: whereas boys’ BMI tends to decrease under the influence of maternal control, girls’ BMI tends to increase [51]. The translation and back-translation process was well conducted, as demonstrated by the ICC values. Results from the Brazilian population can be compared with those from the American population because the Portuguese version shows excellent reproducibility.

The precepts of sDOR are used in public health for food and nutrition education in early childhood [52]. Brazilian legislation establishes that there must be, in the curriculums of preschools and schools, the insertion of food and nutritional education to teach about healthy eating practices and habits. These policies also foresee the involvement of families and the school community, aiming for a wider reach and impact of health promotion initiatives [53]. It is known that the challenge of protecting and promoting children’s health is complex, and for this, the joint engagement of families, schools, and the government is efficient [54]. Policies focusing on educating children’s caregivers about respectful eating practices that also consider the behavioral aspects of eating [54,55], and the precepts established by sDOR [13], can be positive. Nutritional interventions focused on behavioral approaches effectively improve the child’s feeding quality [56]. This reinforces the importance of the results of our study as a starting point in the creation of nutritional education policies for early childhood, taking into account the family and the educational environment where caregivers are inserted. 

It is worth remembering that the present study aimed to translate and perform a psychometric analysis of the instrument, collecting data in a pilot study with a limited sample. Caution is needed when extrapolating the data, because studies with larger samples are needed to use the results in public policy formation. Recognizing that our study has limitations and that caution should be exercised when interpreting the results is important. Some biases can be perceived because it is an online survey with a self-administered instrument with a non-probabilistic convenience sample. It is essential to mention that previous studies on this subject have also used this method, and that if we had used random probabilistic sampling, our sample might be lower than what was achieved in this study. Furthermore, data collection during the COVID-19 pandemic relied on the internet as the primary way to recruit participants due to social interaction limitations. The online spread of the instrument allowed a wider distribution among Brazilian caregivers. It resulted in a larger sample and unidentified participants. Anonymity can reduce the bias associated with the discomfort or shame of reporting data, allowing more accurate responses. The heterogeneous sample composed mainly of female subjects with good income and education levels is also considered a potential bias, as it does not reflect all Brazilians. Furthermore, although data are presented on the reliability of the questionnaire, no data were presented on its validity in this population. Future studies are needed to minimize the abovementioned limitations and to evaluate the Brazilian population in a large and more representative sample.

## 5. Conclusions

The translation of the sDOR.2-6y™ into Brazilian Portuguese is the first and only tool available for the Brazilian population to assess the division of responsibility in feeding. This study showed good results, exhibiting excellent reproducibility. It is worth noting that the evaluation of the instrument’s internal consistency revealed similar values to those found in the original instrument. Further studies are needed on the external nationwide validation of this instrument in Brazil. We emphasize the importance of the sDOR.2-6y™—Portuguese-Brazil for the academic community and for research on child feeding because it is an instrument that can predict nutritional risk in children and point to caregiver behaviors compatible with responsive feeding practices acting in the protection and promotion of children´s health. Its application can reveal important data to be used by health professionals and authorities involved in developing public policies related to child feeding and eating behavior.

## Figures and Tables

**Table 1:** Reproducibility of the sDOR.2-6y™—Português-Brasil by domains and in total (*n* = 23 subjects).

sDOR.2-6y™ Domains	Tests Means (SD ^1^)	Retest Means (SD)	ICC ^2^
Mealtime structure	4.61(1.16)	4.57 (1.08)	0.894
What is available to the child	3.70 (1.89)	3.87 (1.77)	0.814
How food is available to the child	6.09 (1.12)	6.13 (1.01)	0.832
Respect for child autonomy in eating	5.09 (1.59)	5.48 (1.20)	0.700
Who controls what, when, and how much is eaten	3.96 (1.40)	4.00 (1.21)	0.825
Total	23.43 (4.18)	24.04 (3.71)	0.945

^1^ Standard Deviation; ^2^ Intraclass Correlation Coefficient (ICC).

**Table 2:** Characteristics of the individuals (*n* = 384 subjects from the Federal District in Brazil).

		Frequency Sample = 384	%
Caregiver’s gender	Female	325	84.6%
	Male	59	15.4%
Caregiver’s age	21 to 30 years	18	4.7%
	31 to 40 years	275	71.6%
	41 to 50 years	83	21.6%
	51 to 60 years	6	1.6%
	61 to 70 years	2	0.5%
Relation to the child	Mother	317	82.6%
	Father	60	15.6%
	Aunt	2	0.5%
	Grandma	5	1.3%
Marital status	Married or common-law marriage	340	88.8%
	Single	22	5.7%
	Divorced	20	5.2%
	Widowed	2	0.5%
Educational level	High School	15	3.9%
	Undergraduate	78	20.3%
	Graduate	291	75.8%
Monthly income ^1^	Up to BRL 3000	14	3.6%
	BRL 3001 to BRL 5000	17	4.4%
	BRL 5001 to BRL 10,000	59	15.3%
	BRL 10,001 to BRL 15,000	76	19.8%
	More than BRL 15,000	186	48.4%
	Prefer not to inform	32	8.3%
Child’s gender	Female	200	52.1%
	Male	184	47.9%
Child’s age	2 years to 2 years and 11 months	109	28.4%
	3 years to 3 years and 11 months	89	23.2%
	4 years to 4 years and 11 months	79	20.6%
	5 years to 5 years and 11 months	58	15.1%
	6 years exactly	49	12.7%

^1^ BRL: Brazilian Real is the official currency of Brazil, and USD 1.00 = BRL 5.33 (15 October 2022).

**Table 3:** sDOR.2-6y^TM^—Português-Brasil scales by domain segregated by sociodemographic characteristics (*n* = 384 subjects from the Federal District in Brazil).

	D1	D2	D3	D4	D5	Total
	Mean (SD)	Mean (SD)	Mean (SD)	Mean (SD)	Mean (SD)	Mean (SD)
Caregiver’s gender *						
Female (*n* = 325)	4.52 (1.10) ^A^	2.57 (1.41) ^A^	5.90 (1.36) ^A^	2.07 (1.38) ^A^	4.96 (1.92) ^A^	20.02 (3.53) ^A^
Male (*n* = 59)	4.49 (1.10) ^A^	2.64 (1.46) ^A^	5.59 (1.54) ^A^	2.20 (1.23) ^A^	5.24 (2.01) ^A^	20.17 (3.85) ^A^
*p*	0.871	0.709	0.120	0.490	0.317	0.766
Caregiver’s age *						
Up to 40 years (*n* = 293)	4.50 (1.08) ^A^	2.56 (1.38) ^A^	5.85 (1.36) ^A^	2.08 (1.37) ^A^	4.96 (1.85) ^A^	19.95 (3.47) ^A^
More than 40 years (*n* = 91)	4.55 (1.16) ^A^	2.65 (1.53) ^A^	5.86 (1.49) ^A^	2.14 (1.30) ^A^	5.14 (2.18) ^A^	20.34 (3.90) ^A^
*p*	0.718	0.602	0.965	0.678	0.437	0.361
Relation to the child *						
Mother (*n* = 317)	4.51 (1.10) ^A^	2.56 (1.40) ^A^	5.90 (1.36) ^A^	2.06 (1.39) ^A^	4.98 (1.92) ^A^	20.02 (3.54) ^A^
Other (*n* = 67)	4.52 (1.09) ^A^	2.69 (1.47) ^A^	5.61 (1.49) ^A^	2.22 (1.19) ^A^	5.10 (1.99) ^A^	20.15 (3.76) ^A^
*p*	0.939	0.501	0.120	0.378	0.644	0.787
Marital status *						
Married or common-law marriage (*n* = 340)	4.51 (1.11) ^A^	2.56 (1.41) ^A^	5.87 (1.38) ^A^	2.09 (1.35) ^A^	5.07 (1.95) ^A^	20.10 (3.61) ^A^
Others (*n* = 44)	4.57 (1.00) ^A^	2.77 (1.43) ^A^	5.70 (1.49) ^A^	2.09 (1.39) ^A^	4.48 (1.72) ^A^	19.61 (3.24) ^A^
*p*	0.724	0.339	0.456	0.999	0.054	0.399
Educational level *						
High School/Undergraduate (*n* = 93)	4.69 (1.03) ^A^	2.76 (1.36) ^A^	5.78 (1.29) ^A^	2.10 (1.23) ^A^	4.91 (1.67) ^A^	20.25 (3.47) ^A^
Graduate (*n* = 291)	4.46 (1.12) ^A^	2.52 (1.43) ^A^	5.87 (1.42) ^A^	2.09 (1.40) ^A^	5.03 (2.01) ^A^	19.98 (3.61) ^A^
*p*	0.078	0.152	0.596	0.963	0.602	0.524
Monthly income ^1,^**						
Up to BRL 5000 (*n* = 31)	4.84 (0.86) ^A^	3.13 (1.57) ^A^	5.77 (1.31) ^A^	2.16 (1.34) ^A^	5.13 (1.75) ^A^	21.03 (3.74) ^A^
BRL 5001 to BRL 10,000 (*n* = 59)	4.54 (0.99) ^A^	2.66 (1.25) ^A^	5.85 (1.40) ^A^	1.93 (1.35) ^A^	5.14 (1.91) ^A^	20.12 (3.13) ^A^
BRL 10,001 to BRL 15,000 (*n* = 76)	4.66 (1.17) ^A^	2.76 (1.33) ^A^	5.87 (1.32) ^A^	2.07 (1.43) ^A^	5.32 (2.02) ^A^	20.67 (3.58) ^A^
More than BRL 15,000 (*n* = 186)	4.38 (1.08) ^A^	2.44 (1.47) ^A^	5.83 (1.46) ^A^	2.11 (1.31) ^A^	4.87 (1.87) ^A^	19.62 (3.55) ^A^
Prefer not to inform (*n* = 32)	4.59 (1.36) ^A^	2.28 (1.30) ^A^	6.00 (1.22) ^A^	2.28 (1.49) ^A^	4.72 (2.25) ^A^	19.88 (4.06) ^A^
*p*	0.129	0.052	0.973	0.815	0.403	0.116
Child’s gender *						
Female (*n* = 200)	4.55 (1.07) ^A^	2.68 (1.45) ^A^	5.84 (1.43) ^A^	2.15 (1.37) ^A^	4.78 (2.00) ^A^	20.00 (3.81) ^A^
Male (*n* = 184)	4.48 (1.13) ^A^	2.47 (1.37) ^A^	5.86 (1.35) ^A^	2.03 (1.34) ^A^	5.25 (1.84) ^B^	20.09 (3.30) ^A^
*p*	0.553	0.152	0.865	0.376	0.017	0.790
Child’s age **						
2 years (*n* = 109)	4.34 (1.21) ^A^	2.60 (1.51) ^A^	6.00 (1.41) ^A^	2.07 (1.41) ^A^	5.14 (1.73) ^A^	20.15 (3.50) ^A^
3 years (*n* = 89)	4.56 (1.16) ^A^	2.55 (1.42) ^A^	5.78 (1.29) ^A^	2.20 (1.24) ^A^	5.18 (1.93) ^A^	20.27 (3.40) ^A^
4 years (*n* = 79)	4.61 (0.98) ^A^	2.59 (1.33) ^A^	5.52 (1.32) ^A^	2.05 (1.38) ^A^	5.00 (1.97) ^A^	19.77 (3.50) ^A^
5 years (*n* = 58)	4.60 (0.99) ^A^	2.64 (1.28) ^A^	6.02 (1.63) ^A^	2.21 (1.47) ^A^	4.88 (2.26) ^A^	20.34 (4.24) ^A^
6 years (*n* = 49)	4.55 (1.04) ^A^	2.51 (1.52) ^A^	6.00 (1.27) ^A^	1.86 (1.27) ^A^	4.55 (1.86) ^A^	19.47 (3.32) ^A^
*p*	0.419 ^A^	0.991	0.114	0.634	0.382	0.632

Note: Categories with less than 30 observations were grouped; * Student’s *t*-test; ** ANOVA with Tukey’s post hoc test. Groups with the same letters (^A,B^) do not differ significantly;^1^ BRL: Brazilian Real is the official currency of Brazil, and USD 1.00 = BRL 5.33 (15 October 2022); D1—Mealtime structure; D2—What is available to the child; D3—How food is available to the child; D4—Parent gives respect to the child´s autonomy in eating; D5—Who controls what, when, and how much is eaten.

## Data Availability

Not applicable.
